# Assessment of the Penn anomaloscope

**DOI:** 10.1364/BOE.559748

**Published:** 2025-09-10

**Authors:** L. Ling, E. Bilgiç, M. Mak, H. H. Smith, N. Strukov, J. D. Mollon, M. V. Danilova

**Affiliations:** 1 Department of Psychology, University of Cambridge, Downing St., Cambridge CB2 3EB, United Kingdom; 2 Ludwig-Maximilians-Universität München, Department of Psychology, Neuro-Cognitive Psychology, Leopoldstraße 13, 80802 Munich, Germany; 3 Trinity College, Cambridge CB2 1TQ, United Kingdom

## Abstract

The main forms of normal and anomalous human color vision can be classified by the Rayleigh match – the ratio of red and green light that matches an amber reference light. We have used a new device – the Penn Anomaloscope – to obtain Rayleigh matches for a group of normal and anomalous participants. The Penn anomaloscope exhibited a high test-retest reliability, not only giving the same diagnosis for normal and anomalous observers on different occasions but also preserving individual differences among normal observers. There was good agreement with the diagnoses given by an established commercial anomaloscope, the Oculus HMC. Using the DeMarco-Smith-Pokorny theoretical cone sensitivities for anomalous and normal observers, and our own measurements of the spectral power distributions of the primaries of the Penn anomaloscope, we modeled the settings that would be predicted for protanomalous, deuteranomalous, and normal participants. There was close agreement between the settings expected from the modeling and the settings independently obtained empirically. The Penn device is compact and portable, and it may be recommended for field studies and educational purposes.

## 1. Introduction

The Rayleigh match offers a definitive classification of variant forms of color vision [1–3]; and anomaloscopes – refinements of Lord Rayleigh’s ‘colour mixing box’ [4] – are the definitive instruments for measuring the match [5,6]. Anomaloscopes, however, are expensive: Commercial instruments typically command a price that is one or two orders of magnitude greater than that of a new set of Ishihara pseudoisochromatic plates. So color scientists and ophthalmological clinicians will welcome the instrument introduced by David Brainard and his colleagues, which can be constructed for less than $ 100, is very compact, and is readily portable. A brief description of the device has been given by Turner and colleagues [7] and more details are available from the Brainard laboratory [8].

**Fig. 1.**
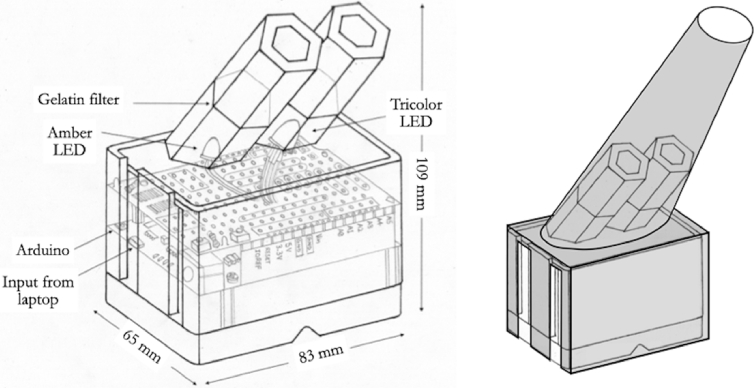
Left: A sketch of the Penn Anomaloscope, showing some details of the interior. Right: Arrangement of added viewing hood.

The Pennsylvania instrument (Fig. 1) can be controlled by a laptop computer via an Arduino microprocessor. LED sources provide the three independent primary lights that any anomaloscope requires. Much of the cost saving is achieved by foregoing the optics that provide a split field in many traditional ophthalmoscopes [5,9–11]: Instead, red/green diffused light from a tricolor LED illuminates one hexagonal field and diffused light from an amber LED illuminates a second, spatially separate field. A prototype of the instrument was generously made available to us by Dr. Brainard.

We report here: (i) the test-retest reliability of the prototype Penn Anomaloscope for normal, protanomalous, and deuteranomalous observers; (ii) the relationship between participants’ settings on the Penn instrument and their settings on a commercial anomaloscope (the Oculus HMC); and (iii) the relationship between the average settings of our three types of observer and the settings expected theoretically from the physical primaries of the instrument.

For the third of these purposes – the modeling of our observers’ settings – we needed estimates of the spectral sensitivities of the cones of the normal, protanomalous and deuteranomalous observers. We used the estimates of DeMarco, Smith and Pokorny [12]. Their normal observer has the color-matching properties of the Judd_1951_ 2-deg Observer as (minimally) modified by Vos [13]. Their anomalous observers were constructed to differ from the Judd_1951_ Observer in either the middle-wave or the long-wave photopigment and to exhibit the average Rayleigh matches of protanomalous and deuteranomalous observers in the large cohort of young military recruits tested by Ingeborg Schmidt [14]. To estimate the sensitivity of the anomalous photopigment of the protanomalous observer, DeMarco *et al.* removed lens and macular absorption from the normal middle-wave photopigment, shifted this template on a frequency abscissa, and then replaced the pre-receptoral factors [12]. The shift was adjusted in small steps to give the best fit to the average Rayleigh match of Schmidt’s protanomalous observers. An analogous procedure was used to estimate the spectral sensitivity of the deuteranomalous photopigment.

It is known, of course, that there is no single protanomalous observer and no single deuteranomalous observer, just as there is no single normal observer [e.g. 9, 15–17]: In all cases, there is likely to be variation in the amino-acid sequence of the opsins [e.g. 18, 19–22], in the effective optical density of the cones [23–25], and in pre-receptoral absorptions [26,27] (although variation in macular pigment should have little effect in the Rayleigh region of the spectrum). In particular, reductions in the optical density of the cones may occur in anomalous observers if some hybrid photopigments are less stable than are the normal ones [25,28]. Even when the sequences of the X-chromosome array are known from genetic analysis, Rayleigh match mid-points cannot be predicted with precision [17, Table 2].

Given the uncertainties in estimating individual cone pigments and given that the DeMarco *et al.* fundamentals were explicitly constructed to yield the average Rayleigh matches of anomalous observers, we judge that these fundamentals are satisfactory for our present purpose. It is relevant that ‘extreme anomalous’ observers – those whose matching range included one end of the anomaloscope range – were excluded from Schmidt’s sample [14]. That was also the case for the present study.

## 2. Methods

### 2.1. Participants

The 32 participants (ten female) were predominantly students or staff of Cambridge University, recruited through social media. Their average age was 27.2 years. Eight participants were anomalous trichromats and the remainder had normal color perception.

Participants were screened and classified by means of the Ishihara pseudoisochromatic plates (9^th^ edition), the Cambridge Colour Test [29,30] and the Oculus HMC Anomaloscope (Oculus Optikgeräte Gmbh, Wetzlar, Germany) [31]. The Ishihara plates were presented under a Macbeth daylight lamp [32].

To confirm their phenotype, anomalous trichromats were additionally tested with the OSCAR test [33,34], which uses a modified form of flicker photometry (‘counterphase modulation photometry’) to measure relative sensitivity to red and green light. We excluded extreme anomalous observers, i.e. those whose settings on the Oculus Anomaloscope extended to one or other extreme of the range.

All participants gave informed written consent. The experiments were approved by the Psychology Research Ethics Committee of Cambridge University (PRE.2021.84).

### 2.2. Equipment

Details of the components and construction of the Penn Anomaloscope are available from the website of the Brainard Laboratory [8]. The compact, 3D-printed, housing (Fig. 1) measures only 83 × 65 × 109 mm. It holds an Arduino ‘Leonardo’ microprocessor and an Adafruit Proto Shield for Arduino (V:R3). The latter carries the two LEDs. Above each LED is a tube that presents a diffuse hexagonal stimulus to the eye. Filters can be mounted in these tubes to modify the spectral power distributions in the two beams.

The red and green primaries are provided by a diffuse tricolor LED and have nominal wavelengths 623 nm and 523 nm respectively (Adafruit component ID: 848). The original amber primary LED had a nominal peak wavelength of 605 nm (Adafruit component ID: 3260) and this LED, like the tricolor LED, was screened with an orange filter (Rosco Roscolux #23). During assembly, we broke the leg of the amber LED and were unable to source an identical replacement. We therefore took the opportunity to substitute an AlGaInP LED (RS Components, UK, 826-717), which has a peak wavelength of 592 nm (dominant wavelength 589.4 nm) and thus is spectrally closer to the D line originally used by Rayleigh [4] and to the amber primary of the Nagel and many subsequent anomaloscopes [5,11,35–37]. In addition, this spectral region favors optimal chromatic discrimination [38]. In order to maintain a suitable range of luminous output, we screened the amber LED with a Wratten 15 cut-on ‘Deep Yellow’ gelatin filter, which has a transmission of 90% at 590 nm. We left in place the orange filter in the bicolor channel.

We made direct calibrations of the three primary outputs of the Penn Anomaloscope, using a JETI spectroradiometer model Specbos 1201 (JETI Technische Instrumente GmbH, Jena, Germany): We measured, in energy units, the peak wavelengths and spectral power distributions of the three primaries, as well as the spectral power distributions of the red/green mixtures over a range of settings of the mixture field. We made analogous measurements for the Oculus Anomaloscope, but using a PhotoResearch spectroradiometer PR670, since this instrument allowed us to focus through the optics of the anomaloscope and select the half-field that we wished to measure.

Whereas in the Nagel or the Oculus anomaloscopes the monochromatic and the mixture fields form two abutting hemicircles, the Penn Anomaloscope presents to the observer two hexagonal fields, which are spatially separated (Fig. 1). To standardize the viewing distance on the Penn instrument, to exclude stray light, and to ensure that viewing was monocular, we constructed a simple conical viewing hood from black card and mounted it on the upper face of the anomaloscope. The viewing distance was approximately 200 mm. At that distance, each hexagonal field subtended 3.29 deg. The inner edges of the two fields were horizontally separated by approximately 3.7 deg.

### 2.3. Procedures

Participants were tested on the Penn and the Oculus Anomaloscopes during the first visit to the laboratory. In both cases, participants viewed the stimuli monocularly with their preferred eye and while wearing their normal spectacle correction as appropriate. All participants made a second visit to the laboratory and gave a second set of settings on the Penn Anomaloscope. On each visit, at least three independent settings were made on the Penn Anomaloscope.

We used the software supplied with the Penn Anomaloscope. We added a key press to the software to save parameters of each adjustment – time; red-green mixture value (designated ‘λ’ in the software); and intensity value for the amber field. A second added key press served to randomize the values of the red-green and amber primaries before each new adjustment.

In order to standardize the participants’ adaptation, they were tested under ambient illumination that had a chromaticity close to Illuminant D65 (Northern Daylight); but during settings on the Penn Anomaloscope the viewing hood (see above) minimized stray light. During testing on the Oculus Anomaloscope, the in-built white field alternated with the test fields.

### 2.4. Modeling

**Fig. 2.**
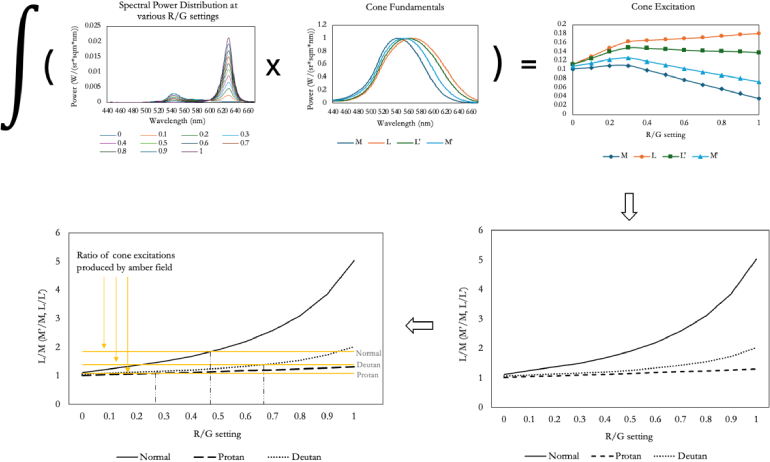
The stages in modeling the settings expected from different types of observer when they make Rayleigh matches on the Penn and the Oculus HMC Anomaloscopes. The spectral power distributions at different R/G settings are multiplied by the normal or by the anomalous cone sensitivites and the products are summed, in order to estimate how the total excitation of each kind of cone varies with R/G setting (top right panel). The ratios of cone excitations are next calculated as a function of R/G (bottom right). The fixed ratios produced by the amber field are represented by horizontal lines in the plot at the bottom left; and the intersection of these lines with the curves for the mixture field give the Rayleigh match that is predicted (vertical broken lines). Note: DeMarco, Pokorny and Smith [12] denote as L’ the anomalous photopigment of the protanomalous and as M’ the anomalous photopigment of the deuteranomalous. Modern usage has reversed this convention [17,28,39] and so we here use L’ for the variant long-wave pigment thought to be present in the deuteranomalous and M’ for the variant middle-wave pigment of the protanomalous.

In order to compare our results with those that might theoretically be expected, we modeled, for the two instruments, the settings predicted from the normal, protanomalous and deuteranomalous observers of DeMarco, Smith and Pokorny [12]. Figure 2 represents the modeling process. To calculate the Rayleigh match that each type of observer would be expected to accept, we first multiplied our empirically measured spectral power distributions at a range of R/G settings by the tabulated cone sensitivities of each of the three observers of DeMarco *et al*. We summed the products to obtain the total excitation of each cone type as a function of R/G settng (top right panel of Fig. 2). The same calculations were performed for the amber primary. (The short-wave cones are omitted from these calculations, since they are minimally sensitive in the Rayleigh range.) We then calculated the *ratios* of cone excitations that would be produced at each value of λ (bottom right panel). In the case of normal observers, these are the ratios L/M. For the protanomalous DeMarco observer, the ratio corresponds to M'/M, and for the deuteranomalous, to L/L’. We similarly calculated the fixed ratio that would be elicited by the amber primary for each type of observer: These are the horizontal lines in the bottom left panel of Fig. 2 and are theoretically independent of the intensity of the amber primary. We then found the value of λ that the observer would be expected to choose in order to match the ratio produced by the amber primary: Graphically, this corresponds to the intersection of the pairs of functions in the bottom left panel. We made analogous calculations for the Penn and the Oculus instruments.

## 3. Results and discussion

### 3.1. Spectral power distributions

**Fig. 3.**
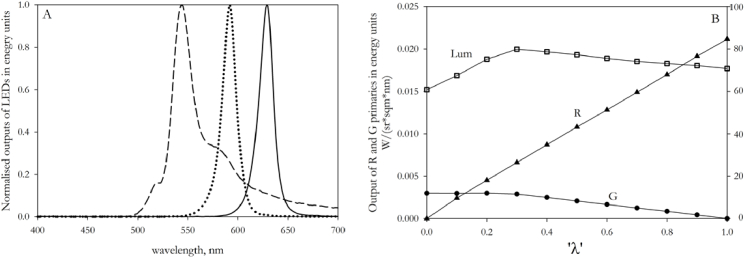
**A.** The normalized spectral power distributions of individual primary lights of the Penn Anomaloscope used in the present study. **B**. The intensities of the red and green primaries, and the total luminous output, at different values of the variable ‘λ’, which represents the setting of the red-green mixture. The circles represent the value of the green primary and the triangles represent the red primary. The squares represent the total luminous output of the mixture field.

Figure 3(A) shows the normalized spectral power distributions of the three primaries of the Penn Anomaloscope. The green primary – one of the outputs of the tricolor LED – has a shoulder at long wavelengths. In Fig. 3(B) we show how the intensities of the red and green primaries vary in relationship to λ, the indicator of R/G ratio provided by the Penn software: The intensity of the red primary increases almost linearly with λ, but the green primary remains constant until λ=0.3. In consequence, the luminous output of the mixture does not remain constant as λ is varied (open squares in graph).

### 3.2. Evaluation of test-retest reliability on the Penn anomaloscope

**Fig. 4.**
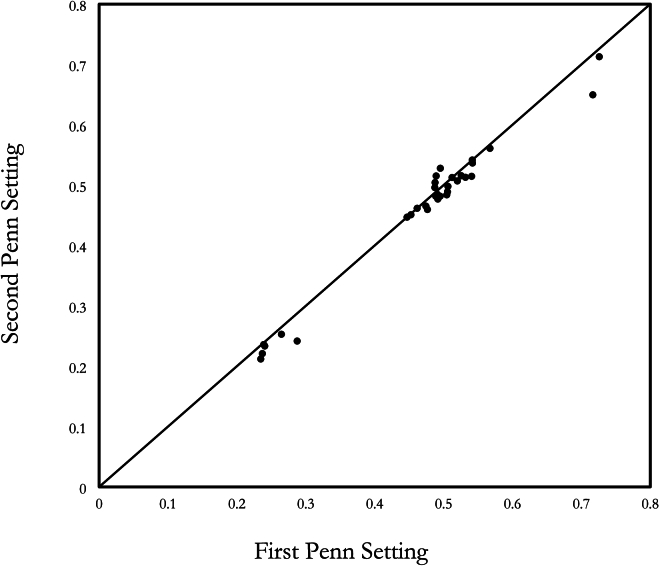
The test-retest reliability between the two sessions of repeated settings on the Penn Anomaloscope, as made by normal and anomalous observers.

Figure 4 shows the relationship between the average of the first set of empirical settings for our individual observers and the average of the second set (which were performed on a different day). The ordinates of the graph are the raw readings of the instrument (‘λ’). A Pearson’s correlation test revealed a strong relationship between the two independent settings on the Penn Anomaloscope (*R* = 0.886, *p* < .001) when only normal observers were included. Thus the Penn instrument is precise enough and stable enough to preserve the well-known variation among normal observers. Turner *et al.* [7] have similarly reported that the Penn Anomaloscope exhibits good test-retest reliability for normal observers. When we included anomalous trichromats in our sample, there was a very strong relationship indeed between the two settings (*R* = 0.989, *p* < .001).

### 3.3. Comparison of settings on the Penn and the Oculus anomaloscopes

**Fig. 5.**
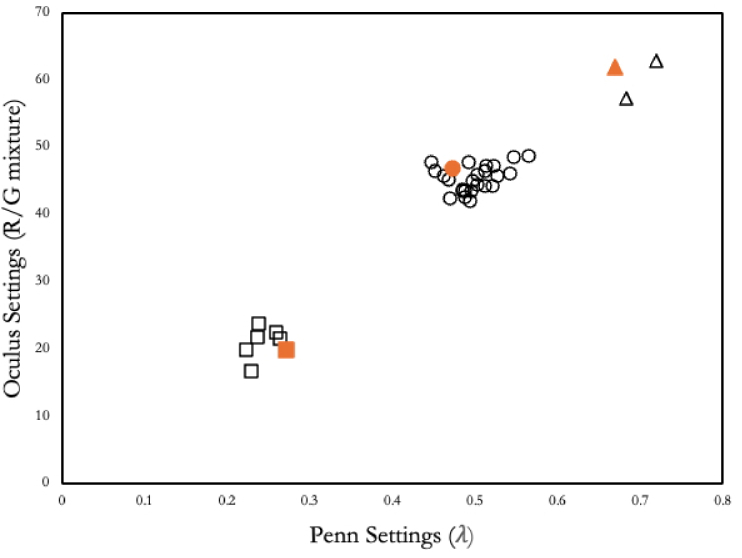
The relationship between settings made on the Penn and on the Oculus instruments. The open symbols are the empirical settings made by our observers. Circles correspond to normal observers, triangles to protanomalous, squares to deuteranomalous. The solid orange symbols are the values that our modeling predicts for the normal, protanomalous and deuteranomalous observers of DeMarco, Smith and Pokorny. The ordinates of the graphs are expressed in terms of the raw scales of the two instruments.

Figure 5 plots, for individual normal, protanomalous, and deuteranomalous observers, the relationship between their empirical settings on the Penn instrument and their corresponding settings on the Oculus (open symbols). The settings are expressed in the units of the raw readings of the two instruments (λ in the case of the Penn device, and the R/G mixture scale (0–73) in the case of the Oculus). We do not express the ordinates in anomalous quotients, since the latter are intended only for comparing instruments with the same primaries [1,36].

The empirical settings show a good overall agreement: In no case did the two instruments offer contradictory diagnoses, or a diagnosis that contradicted our secondary measures (Cambridge Colour Test; OSCAR test). When anomalous trichromats are included in the total sample, there is a very strong relationship between empirical measures on the two instruments: The value of Pearson’s correlation is *R* = 0.971 (*p* < .001). The value for the non-parametric Spearman correlation ρ (which does not assume that the underlying distributions are normal) was 0.728 (*p* < .001). If only the population of normal observers is considered, the Pearson correlation is positive but non-significant (*R* = 0.356, *p* = 0.088) and Spearman’s ρ is 0.361 (*p* = .084).

Although a linear fit to the empirical data of Fig. 5 would capture much of the variance, there is no *a priori* reason to expect a linear relationship. There are two reasons for this. Firstly, owing to the way that the output of the green primary of the Penn device varies with λ (Fig. 3(b)), the relationship between λ and R/G is non-linear. Secondly, there are large differences in the primary wavelengths of the two anomaloscopes. Owing to observer metamerism [40], there is no common metric for comparing individual settings on anomaloscopes with different primaries. Within both the anomalous and the normal groups, each observer has his or her own set of cone sensitivities, and these would need to be multiplied by the two different sets of primaries in order to estimate the relative excitations of the cones in each case.

### 3.4. Modeling

Also shown in Fig. 5 (solid symbols) are the average settings expected theoretically for the three types of DeMarco-Smith-Pokorny observers. These settings were modeled as described in Methods and are independent of the empirical settings of the current participants. The modeled values in Fig. 5 depend only on the tabulated sensitivities of the DeMarco observers and on our physical measurements of the outputs of the two anomaloscopes. The agreement is good. We would not expect perfect agreement, since our average observers would not be expected to coincide exactly with the DeMarco-Smith-Pokorny observers and since there must be residual experimental error in the settings of our observers and in our calibrations. It is relevant, of course, that anomalous observers used in the modeling [12] were explicitly constructed to have the average settings that were made by Schmidt’s anomalous observers on a Nagel anomaloscope with primaries 537, 589 and 666 nm [14].

### 3.5. Limitations

Ideally, the long-wavelength primary of the Penn Anomaloscope would be moved from 623 nm to be close to the classical Nagel primary (typically 665–670 nm): The greater the separation of the primaries, and the narrower their bandwidths, the better will an anomaloscope separate different phenotypes [3,9,36]. However, the present long-wave primary is constrained by the availability of suitable tricolor LEDs (as was the case for a solid-state anomaloscope developed 40 years ago by Kintz [41]). The large advantage of the use of a tricolor LED for the red and green primaries is that no optics are required to produce a reasonably uniform mixing field: This factor is critical to the low cost and the compactness of the Penn Anomaloscope. Another promising new, low-cost anomaloscope is similarly limited by the primaries available [42]. It would, however, be readily possible to move the amber primary of the Penn device to a wavelength closer to the 589 nm of the Nagel anomaloscope – as we have done in the present study.

One property of the Penn device is that the monochromatic and mixture fields cannot be juxtaposed as semi-circular half fields, as they are in the classical anomaloscope of Nagel [5] or in many subsequent instruments such as the Oculus [31]. These different spatial arrangements offer a further reason why we would not expect a perfect relationship between the Penn and the Oculus Anomaloscopes. Nevertheless, the spatial separation of the two fields on the Penn instrument may not degrade the observer’s performance: Separation of the discriminanda does not necessarily impair color discrimination and a small separation may even enhance it [43–46].

## 4. Conclusions

The Penn Anomaloscope is compact and portable enough to be used in field research outside the laboratory. It is economical to construct. Yet it exhibits good test-retest reliability, its diagnoses are concordant with those obtained with the Oculus HMC Anomaloscope and with other tests, and its results are theoretically valid. The Penn instrument would certainly be satisfactory for routine screening and diagnosis. However, it would at present be inappropriate to use it in any situations where the measurements may have legal implications, such as testing for entry to professions that have formal standards for color vision. In particular, the device would not satisfy the German standard for anomaloscopes, DIN 6160:2019, which specifies wavelengths close to those of the classical Nagel anomaloscope [2].

The Penn Anomaloscope would especially recommend itself for teaching, for example, in schools of medicine or optometry or psychology; for it would be feasible to provide enough devices for all members of the class to have extended experience in using the instrument. Indeed, our experience suggests that some students may enjoy constructing the Penn Anomaloscope using the instructions provided by its designers.

## Data Availability

Data underlying the results presented in this paper are not publicly available at this time but are available from the authors upon reasonable request.

## References

[1] Pokorny J. Smith V. C. Verriest G. et al., *Congenital and Acquired Color Vision Defects* (Grune & Stratton, 1979).

[2] Dain S. J. Hovis J. K., “Recommendations and requirements for the wavelengths in Rayleigh equation anomaloscopes,” J. Opt. Soc. Am. A 40(3), A121–A129 (2023). 10.1364/JOSAA.477144 37133022

[3] Pokorny J. Smith V. C., “Metameric matches relevant for assessment of color vision,” in *Colour Vision Deficiences VII (Documenta Ophthalmologica Proceedings Series* vol 39), Verriest G., eds. (Dr W Junk Publishers, 1984), pp. 83–94.

[4] Rayleigh L., “Experiments on colour,” Nature 25(629), 64–66 (1881). 10.1038/025064a0

[5] Nagel W. A., “Zwei Apparate für die Augenärzliche Funktionsprüfung. Adaptometer und kleines Spektralphotometer (Anomaloskop),” Ophthalmologica 17(3), 201–222 (1907). 10.1159/000291204

[6] Mitchell D. E. Rushton W. A. H., “Red/Green Pigments of Normal Vision,” Vision Res. 11(10), 1045–1056 (1971). 10.1016/0042-6989(71)90111-8 5316543

[7] Turner D. Keesing L. Gray J. et al., “Testing the reliability and validity of Rayleigh matches and heterochromatic flicker photometry settings on an Arduino-based LED device,” in *27th International Colour Vision Society Meeting* , Tekavčič Pompe M., eds. (University Eye Clinic, Ljubljana, 2024), pp. 59–60.

[8] Keesing L. Brainard D., “The Penn anomaloscope https://github.com/BrainardLab/PennAnomaloscope.”

[9] Willis M. P. Farnsworth D., Comparative evaluation of anomaloscopes, (Medical Research Laboratory, Bureau of Medicine and Surgery, US Navy, 1952).

[10] Moreland J. D. Young W. B., “A new anomaloscope employing interference filters,” Mod. Probl. Ophthal 13, 47–55 (1974). 4437547

[11] Pokorny J. Smith V. C. Lutze M., “A computer-controlled briefcase anomaloscope,” in *Colour Vision Deficiencies IX (Documenta Ophthalmologica Proceedings Series 52)* , Drum B. Verriest G., eds. (Kluwer Academic, 1989), pp. 515–522.

[12] DeMarco P. Pokorny J. Smith V. C., “Full-Spectrum Cone Sensitivity Functions for X-Chromosome-Linked Anomalous Trichromats,” J. Opt. Soc. Am. A 9(9), 1465–1476 (1992). 10.1364/JOSAA.9.001465 1527649

[13] Vos J. J., “Colorimetric and photometric properties of a 2 deg fundamental observer,” Color Res. Appl. 3(3), 125–128 (1978). 10.1002/col.5080030309

[14] Schmidt I., “Some problems related to testing color vision with the Nagel anomaloscope,” J. Opt. Soc. Am. 45(7), 514–522 (1955). 10.1364/JOSA.45.000514 14381950

[15] Alpern M. Pugh E. N., “Variation in Action Spectrum of Erythrolabe among Deuteranopes,” J Physiol-London 266(3), 613–646 (1977). 10.1113/jphysiol.1977.sp011785 301186 PMC1283583

[16] Alpern M., “The Color-Vision of the Color Blind,” Jpn. J. Ophthalmol. 25, 1–17 (1981).

[17] Bosten J., “The known unknowns of anomalous trichromacy,” Curr Opin Behav Sci 30, 228–237 (2019). 10.1016/j.cobeha.2019.10.015

[18] Winderickx J. Lindsay D. T. Sanocki E. et al., “Polymorphism in red photopigment underlies variation in colour matching,” Nature 356(6368), 431–433 (1992). 10.1038/356431a0 1557123

[19] Sharpe L. T. Stockman A. Jagle H. et al., “Red, green, and red-green hybrid pigments in the human retina: Correlations between deduced protein sequences and psychophysically measured spectral sensitivities,” J. Neurosci. 18(23), 10053–10069 (1998). 10.1523/JNEUROSCI.18-23-10053.1998 9822760 PMC6793300

[20] Neitz J. Neitz M., “The genetics of normal and defective color vision,” Vision Res. 51(7), 633–651 (2011). 10.1016/j.visres.2010.12.002 21167193 PMC3075382

[21] Asenjo A. B. Rim J. Oprian D. D., “Molecular determinants of human red/green color discrimination,” Neuron 12(5), 1131–1138 (1994). 10.1016/0896-6273(94)90320-4 8185948

[22] Sanocki E. Teller D. Y. Deeb S. S., “Rayleigh match ranges of red/green color-deficient observers: Psychophysical and molecular studies,” Vision Res. 37(14), 1897–1907 (1997). 10.1016/S0042-6989(97)00005-9 9274775

[23] Shevell S. K. He J. C., “The visual photopigments of simple deuteranomalous trichromats inferred from color matching,” Vision Res. 37(9), 1115–1127 (1997). 10.1016/S0042-6989(96)00270-2 9196730

[24] Thomas P. B. Formankiewicz M. A. Mollon J. D., “The effect of photopigment optical density on the color vision of the anomalous trichromat,” Vision Res. 51(20), 2224–2233 (2011). 10.1016/j.visres.2011.08.016 21893078

[25] Renner A. B. Knau H. Neitz M. et al., “Photopigment optical density of the human foveola and a paradoxical senescent increase outside the fovea,” Visual Neuroscience 21(6), 827–834 (2004). 10.1017/S0952523804216030 15733338 PMC2603297

[26] Maxwell J. C., “On colour vision,” Proceedings of the Royal Institution of Great Britain 6, 260–271 (1871).

[27] Webster M. A. MacLeod D. I. A., “Factors underlying individual differences in the color matches of normal observers,” J. Opt. Soc. Am. A 5(10), 1722–1735 (1988). 10.1364/JOSAA.5.001722 3204435

[28] Mollon J. D., “’…aus dreyerley Arten von Membranen oder Molekülen': George Palmer's legacy,” in *Colour Vision Deficiencies XIII* , Cavonius C. R., eds. (Kluwer, Documenta Ophthalmologica Proceedings Series vol. 59, 1997), pp. 3–20.

[29] Regan B. C. Reffin J. P. Mollon J. D., “Luminance noise and the rapid determination of discrimination ellipses in colour deficiency,” Vision Res. 34(10), 1279–1299 (1994). 10.1016/0042-6989(94)90203-8 8023437

[30] Mollon J. D. Reffin J. P., “A Computer-Controlled Color-Vision Test That Combines the Principles of Chibret and of Stilling,” J Physiol-London 414, P5 (1989).

[31] Krastel H. Gehrung H. Dax K. et al., “Clinical-Application of the Heidelberg Anomaloscope,” in *Colour Vision Deficiencies X* , Drum B. Moreland J. D. Serra A., eds. (Kluwer Academic, 1991), pp. 135–149.

[32] Farnsworth D., “Abridgment and administration of the A. O. 1st edition pseudo-isochromatic plates,” in *Color Vision Report No 14 BuMed project X-749* (U.S. Navy Medical Research Laboratory New London, 1946).

[33] Estévez O. Spekreijse H. van Dalen J. T. W. et al., “The Oscar color vision test: theory and evaluation (Objective Screening of Color Anomalies and Reductions),” American Journal of Optometry and Physiological Optics 60(11), 892–901 (1983). 10.1097/00006324-198311000-00004

[34] Jordan G. Mollon J. D., “Sons and mothers: Classification of colour-deficient and heterozygous subjects by counterphase modulation photometry,” Doc. Ophthalmol. Proc. Ser. 59, 385–392 (1997). 10.1007/978-94-011-5408-6_42

[35] Pelizzone M. Sommerhalder J. Roth A. et al., “Automated Rayleigh and Moreland Matches on a Computer-Controlled Anomaloscope,” Colour Vision Deficiencies X 54, 151–159 (1991). 10.1007/978-94-011-3774-4_19

[36] Dain S. J. Hovis J. K., “Modeling wavelengths and spectral half-power bandwidths for Rayleigh equation anomaloscopes,” J. Opt. Soc. Am. A 42(5), B214–B224 (2025). 10.1364/JOSAA.545024 40793527

[37] Piantanida T. P., “A portable filter anomaloscope,” Opt. Eng. 15, 325–327 (1976). 10.1117/12.7971984

[38] Bouman M. A. Walraven P. L., “Color discrimination data,” in *Visual Psychophysics* Jameson D. Hurvich L. M., eds. (Springer 1972).

[39] Boehm A. E. Bosten J. MacLeod D. I. A., “Color discrimination in anomalous trichromacy: Experiment and theory,” Vision Res. 188, 85–95 (2021). 10.1016/j.visres.2021.05.011 34293614

[40] Knoblauch K., “Representing an observer’s matches in an alien colour space,” in *Normal and Defective Colour Vision* , Mollon J. D. Pokorny J. Knoblauch K., eds. (Oxford University Press, 2003), pp. 267–272.

[41] Kintz R. T., “A portable, solid-state anomaloscope,” Behavior Research Methods & Instrumentation 15(6), 587–590 (1983). 10.3758/BF03203728

[42] Rezeanu D. Kuchenbecker J. A. Neitz M. et al., “iPhone-based anomaloscope for accessible, accurate color vision testing,” J. Opt. Soc. Am. A 42(5), B34–B42 (2025). 10.1364/JOSAA.545284 PMC13105152 40793510

[43] Boynton R. M. Hayhoe M. M. MacLeod D. I. A., “The gap effect: chromatic and achromatic visual discrimination as affected by field separation,” Opt. Acta 24(2), 159–177 (1977). 10.1080/713819496

[44] Eskew R. T., “The gap effect revisited: Slow changes in chromatic sensitivity as affected by luminance and chromatic borders,” Vision Res. 29(6), 717–729 (1989). 10.1016/0042-6989(89)90034-5 2626829

[45] Danilova M. V. Mollon J. D., “The comparison of spatially separated colours,” Vision Res. 46(6-7), 823–836 (2006). 10.1016/j.visres.2005.09.026 16288793

[46] Danilova M. V. Mollon J. D., “The gap effect is exaggerated in the parafovea,” Visual Neuroscience 23(3-4), 509–517 (2006). 10.1017/S0952523806233327 16961988 PMC2648725

